# Correction to “Novel approaches in myelofibrosis”

**DOI:** 10.1002/hem3.70222

**Published:** 2025-10-13

Koschmieder S. Novel approaches in myelofibrosis. *HemaSphere*. 2024;8(12):e70056. doi:10.1002/hem3.70056

A funder was not acknowledged in the original article. The following statement has now been added in the Funding section: “Open Access funding enabled and organized by Projekt DEAL.”

The original article has been updated. We apologize for this error.

